# Purely Epidural Thoracic Meningioma With Elevated Ki-67 in a Postmenopausal Female With Prior Gynecologic Malignancies: A Case Report

**DOI:** 10.7759/cureus.90372

**Published:** 2025-08-18

**Authors:** Ryan Lebens, Bayan Razzaq, John Servider, Robert S Kleyner, Mohsen Ahmed, Harry Mushlin

**Affiliations:** 1 Orthopaedic Surgery, Stony Brook University, Stony Brook, USA; 2 Neurological Surgery, Stony Brook University, Stony Brook, USA; 3 Neurosurgery, Stony Brook University, Stony Brook, USA; 4 Internal Medicine, Stony Brook University, Stony Brook, USA

**Keywords:** epidural meningioma, gyne-oncology, hormonal replacement therapy, ki-67 expression, primary spinal tumor

## Abstract

Meningiomas are common central nervous system (CNS) tumors; however, purely epidural spinal meningiomas are an exceedingly rare subtype. Their differentiation from other extradural lesions, particularly in patients with a history of malignancy, remains a diagnostic challenge. Additionally, the potential influence of hormonal factors on meningioma development is not fully understood.

Here, we present the case of a 60-year-old female with a purely epidural thoracic meningioma, notable for its occurrence after a history of stage IA endometrial and ovarian cancer. Despite its classification as a World Health Organization (WHO) Grade 1 meningioma, the tumor exhibited an elevated Ki-67 index of 4.5%, a finding rarely documented in purely extradural spinal meningiomas. This case contributes to the limited literature on extradural meningiomas and raises questions about the biological behavior of such tumors, particularly in patients with prior gynecologic malignancies and hormone replacement therapy exposure.

This case underscores the importance of considering oncologic history and hormonal influences in the evaluation of spinal tumors. It also highlights the diagnostic and management challenges associated with purely epidural meningiomas and emphasizes the potential role of Ki-67 as a prognostic marker in extradural lesions. Further research is warranted to explore the interplay between hormonal factors and meningioma pathogenesis.

## Introduction

Meningiomas account for approximately 37.6% of all central nervous system (CNS) tumors, with 12.7% occurring in the spine [1,2]. These tumors are typically benign, with 90% classified as World Health Organization (WHO) grade I, and they have an incidence rate of 64 per 100,000 person-years [1]. The vast majority of spinal meningiomas are intradural extramedullary tumors, most commonly found in the thoracic region (70%), followed by the cervical spine (25%) and the thoracolumbar region (5%) [3]. Women are disproportionately affected, with a female-to-male incidence ratio of 4:1 [1].

While spinal meningiomas are usually intradural, some extend extradurally, and an even smaller subset presents as purely extradural lesions, occurring in only 2.5%-3.5% of cases [4]. Given their rarity, purely epidural meningiomas may be misdiagnosed as more common spinal neoplasms, including schwannomas, neurofibromas, lymphomas, and metastases [1]. Schwannomas, in particular, share a similar location and slow growth rate, making radiologic differentiation challenging [3].

Here, we present the case of a purely epidural thoracic meningioma in a 60-year-old female with a history of stage IA dual primary endometrial and ovarian endometrioid adenocarcinoma, previously treated with total laparoscopic hysterectomy and bilateral salpingo-oophorectomy. This case is of particular significance due to two key factors: (1) it is amongst the few reported instances of a purely epidural meningioma and the first to exhibit an atypically elevated Ki-67 index (>4%) despite being classified as WHO Grade I, raising questions about its biological behavior, and (2) it adds to the growing body of evidence suggesting a potential link between hormonal factors and meningioma development, especially in patients with a history of gynecologic malignancies. The patient’s extensive oncologic history further highlights the importance of vigilant surveillance and multidisciplinary management in cases of spinal meningioma with a complex systemic background.

## Case presentation

**Figure 1:**
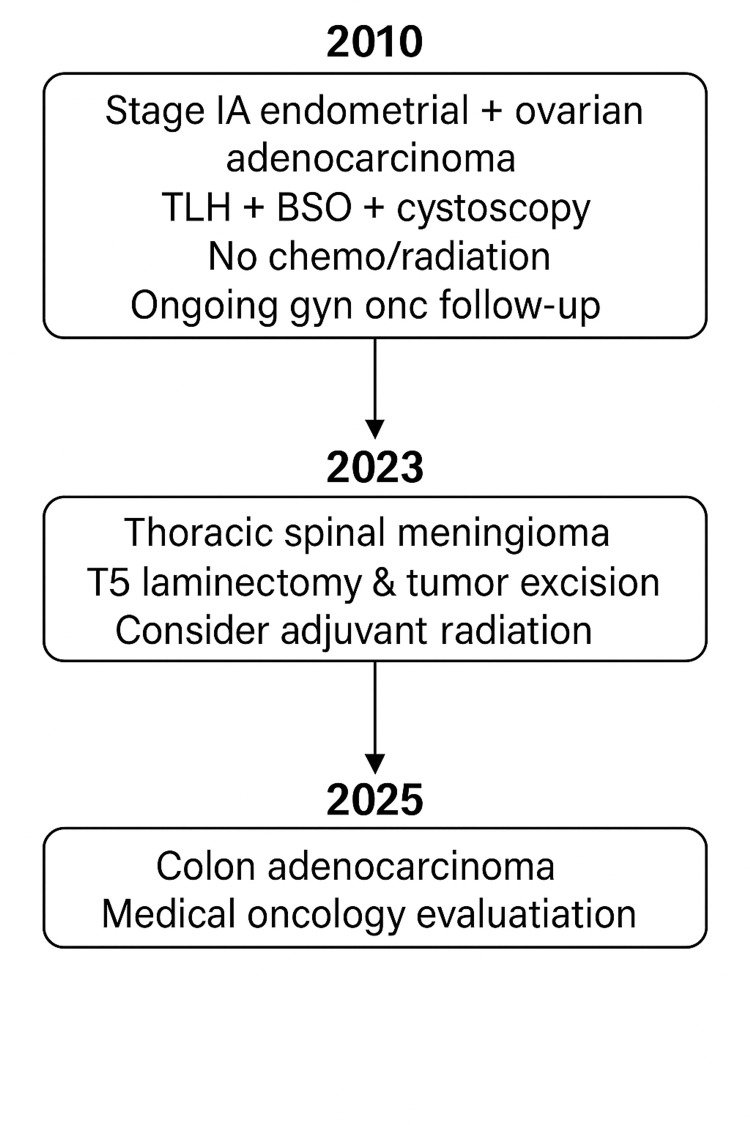
Flowchart of presenting patients’ cancer history and interventions.

A 60-year-old female with a history of ovarian and uterine cancer status post total hysterectomy and bilateral oophorectomy, breast mass excision, epilepsy with grand mal seizures, anti-epileptic drug treatment, migraines, depression, and anxiety presented with six months of progressive rib pain radiating to the anterior thoracic wall. Over time, she developed worsening lower extremity weakness and occasional dizziness, eventually leading to difficulty ambulating. On neurological examination, she exhibited hyperreflexia, bilateral clonus, and a mildly positive Romberg test with a negative pull test. Strength was preserved at 5/5 in all four limbs with no pronator drift, and sensation was grossly intact to light touch throughout. However, her gait was observed to be narrow-based with slightly unsteady, heavy footsteps. Figure 1 shows the flowchart of patients' cancer history and interventions.

**Figure 2:**
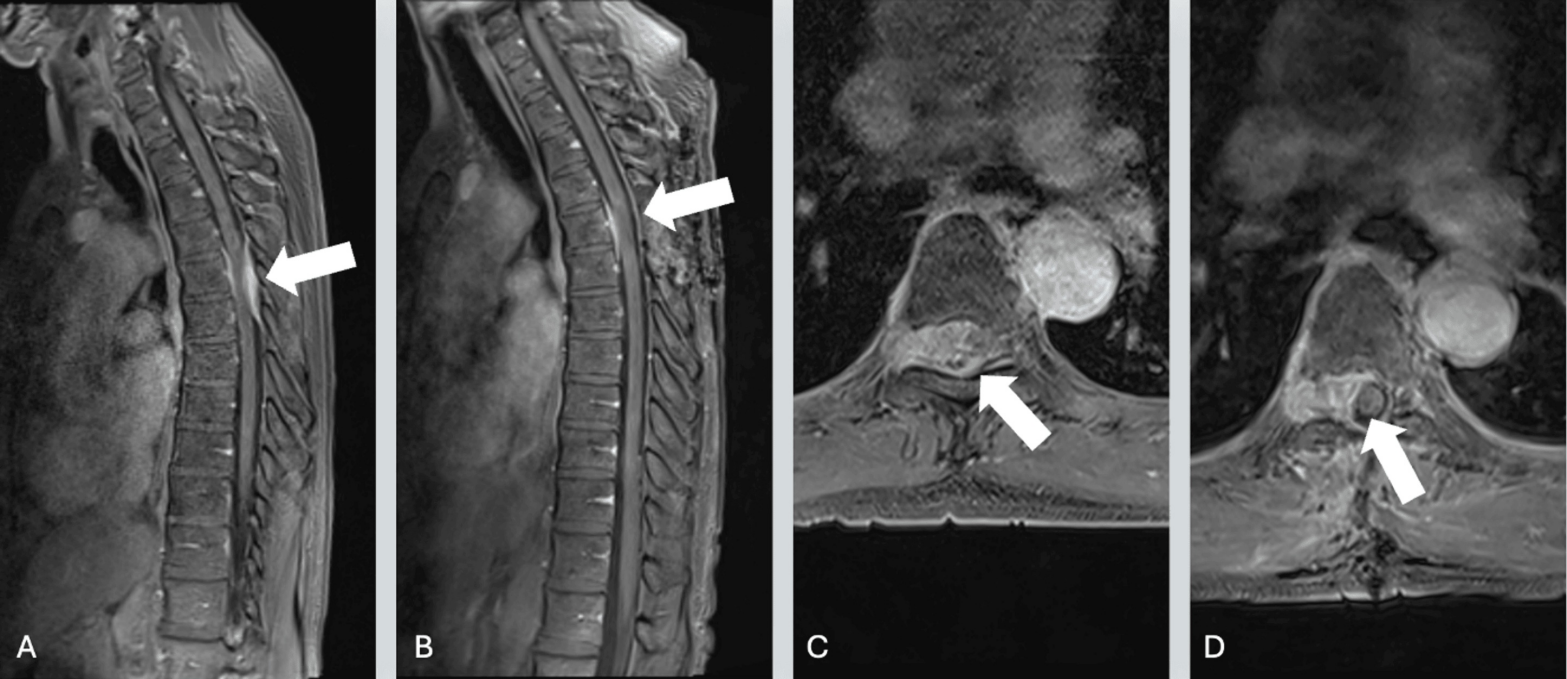
Sagittal magnetic resonance imaging (MRI) of the thoracic spine displaying the lesion at T5-T6 (A) and subsequent postoperative sagittal imaging acquired one day after resection (B). Axial MRI revealing a lesion at the T5-T6 region preoperatively (C) with a subsequent axial MRI one day postoperative (D).

MRI of the thoracic spine revealed an enhancing extradural mass at the T5-T6 level, occupying the right side of the central spinal canal and extending into the right neuroforamina. The lesion displaced the thecal sac and compressed the thoracic spinal cord, with associated increased T2 signal intensity consistent with cord edema (Figure 2). The mass measured approximately 1.3 cm in anteroposterior diameter, 2.48 cm in transverse diameter, and 3.85 cm in height. It demonstrated homogeneous enhancement on post-contrast imaging. No disc protrusions, dural tail sign, vertebral abnormalities, deformities, or spondylolisthesis were noted. Based on its radiologic appearance and location, the initial differential diagnosis included spinal schwannoma and metastatic disease.

The patient was taken emergently for resection of the presumed metastatic epidural tumor. A T5 laminectomy was performed, extending partially to T4 and T6 to expose normal dura. Intraoperatively, the epidural lesion was identified and debulked. The tumor extended into the right T5 foramen, which was also partially debulked. Complete resection was not pursued due to the lesion’s extension into the right T5-T6 neuroforamina, where further dissection posed a risk of nerve root injury. The frozen specimen initially suggested a malignant metastatic process, leading to concern for metastatic disease. However, subsequent histopathological analysis confirmed a CNS WHO grade 1 meningioma.

Postoperatively, the patient remained neurologically intact. She exhibited increased muscle tone in both lower extremities, though sensation remained intact. She denied any bowel or bladder dysfunction. Notably, her preoperative rib pain resolved, and gait instability was no longer present. Two weeks postoperatively, the patient developed new-onset vertigo and a small area of numbness at the left lateral rib area at T5. However, her gait and ambulation remained unaffected. MRI at this time revealed residual meningioma at the right T5 level, and the patient was scheduled for adjuvant radiation therapy.

At the six-month follow-up, the patient reported continued resolution of her myelopathy and radicular pain. However, during this period, she was diagnosed with stage IIB colon cancer and underwent chemoradiation following resection of the colorectal neoplasm. A one-year follow-up MRI demonstrated no significant interval change in the size or appearance of the residual extradural neoplasm. At both the one-year and 18-month follow-ups, the patient remained neurologically stable with resolution of myelopathic and radicular symptoms. Although she was scheduled to receive a single 14-gray dose of radiation therapy for the residual meningioma, this treatment was delayed due to her ongoing colorectal cancer management.

Immunohistochemical staining was positive for Ki-67, epithelial membrane antigen (EMA), and progesterone receptor (PR). Notably, the Ki-67 labeling index was elevated to 4.5% (603 cells counted), an atypical finding for a WHO grade 1 spinal meningioma. No carcinoma was identified in the specimen, confirming the diagnosis of classification of tumors of the central nervous system by World Health Organization (CNS WHO) grade 1 meningioma.

## Discussion

We observe the case of a 60-year-old female presenting with gait difficulties, thoracic rib pain, lower extremity weakness, and a thoracic epidural tumor, later confirmed to be a meningioma. Given the patient’s previous cancer history and intraoperative presentation, metastatic disease was initially highly suspected. As of 2022, there were fewer than 50 reported cases of purely epidural spinal meningiomas, with only five additional documented cases since 2022 [3], [5-9]. Of the documented cases, nearly all were reported as CNS WHO Grade 1 meningioma. None of these cases reported Ki-67 index markers above 4%, making the present case a novel and important contribution to the literature.

Meningiomas are typically benign, with 80.5% classified as CNS WHO Grade 1, while Grade II and III tumors account for 17.7% and 1.7%, respectively [3]. Despite their indolent nature, there is growing recognition that certain biological markers may help stratify tumor behavior and recurrence risk. Among these, the Ki-67 proliferation index has emerged as an important prognostic indicator, with values exceeding 4% associated with more aggressive growth and poorer outcomes in meningiomas [10]. While Ki-67 is frequently used to differentiate higher-grade meningiomas, to our knowledge, no prior reports have documented a purely epidural WHO Grade 1 spinal meningioma with a Ki-67 index greater than 4%. Of note, spinal meningiomas tend to have lower proliferation indices compared to cranial counterparts, making this case of particular interest [10]. Importantly, no other WHO grade II features were observed on histopathology. This case, therefore, challenges conventional assumptions regarding the biological behavior of WHO Grade 1 spinal meningiomas and raises critical questions about whether a subset of these tumors may exhibit more aggressive potential despite their benign histology. Although the long-term implications of elevated Ki-67 in spinal meningiomas remain unclear, its potential role in guiding follow-up intensity and treatment decisions warrants further investigation.

While the hormonal environment has been implicated in meningioma development, no prior reports, to our knowledge, have documented a spinal meningioma occurring after uterine or ovarian cancer and subsequent hysterectomy. The higher incidence of meningiomas in females, with a reported sex ratio ranging from 3:1 to 4:1, suggests a possible hormonal influence on tumor pathogenesis [1]. Additionally, 72.2% of meningiomas express progesterone receptors, supporting the hypothesis that hormonal factors may play a role in both tumor development and progression [11]. Although no studies have directly linked ovarian or uterine cancer to spinal meningiomas, hormone replacement therapy (HRT) has been identified as a potential risk factor. Several studies suggest an association between HRT use and meningioma growth, with some recommending against its use in individuals with known meningiomas [12]. In this case, the patient had intermittently received Estratest as HRT for over a decade prior to her spinal meningioma diagnosis, raising the possibility that prolonged hormonal exposure may have influenced tumor development. Additionally, Yen et al. found that women with a history of uterine myomas had a higher likelihood of developing meningiomas, further suggesting a complex interplay between hormonal signaling and meningioma pathogenesis [13]. While a direct causal relationship cannot be established, this case highlights the need for further research into the potential role of hormonal factors in spinal meningioma development, particularly in patients with prior gynecologic malignancies and HRT exposure.

In addition to hormonal influences, this patient’s history of multiple malignancies, including uterine, ovarian, colorectal, and spinal meningiomas, raises the possibility of an underlying genetic predisposition. Lynch syndrome, characterized by mutations in DNA mismatch repair genes, is known to increase the risk of colorectal, endometrial, and ovarian cancers [14]. Given this patient's oncologic history, Lynch syndrome or another hereditary cancer syndrome warrants consideration. Further genetic evaluation in similar patients may provide insights into potential links between systemic malignancies and meningiomas, particularly in cases where multiple primary neoplasms are present. At the time of publication, genetic testing was in progress. 

This case contributes to the literature in two critical ways. First, it represents the first reported purely epidural spinal meningioma with a Ki-67 index exceeding 4%, expanding the limited understanding of purely extradural spinal meningiomas. While WHO Grade 1 meningiomas are typically considered indolent, the elevated Ki-67 index observed in this case challenges conventional assumptions about their biological behavior. This suggests that even histologically benign spinal meningiomas may exhibit proliferative potential beyond what is typically expected, warranting further investigation into Ki-67 as a prognostic marker in extradural lesions. Additionally, this case underscores the diagnostic challenge of distinguishing epidural meningiomas from other extradural spinal tumors, particularly in patients with a history of malignancy, reinforcing the importance of careful preoperative evaluation and surgical planning. Second, this case highlights the need for closer surveillance in spinal meningioma patients with complex medical histories. While spinal meningiomas are generally regarded as low-risk, the combination of this patient’s prior malignancies and an epidural meningioma with an elevated Ki-67 index suggests that standard follow-up protocols may not always be sufficient. In such cases, ongoing monitoring should account for both the potential for tumor progression and the broader differential diagnosis of spinal lesions in patients with previous cancers. This case reinforces the importance of an individualized approach to follow-up and management in spinal meningiomas, ensuring that both tumor behavior and differential diagnostic considerations are carefully evaluated. In this case, no molecular profiling (e.g., TRAF7, KLF4, SMO, or NF2 mutation testing) was performed. Such analyses could provide additional prognostic insight and help clarify whether distinct genetic alterations contribute to the atypical proliferative index observed. The absence of molecular data represents a limitation of this report.

## Conclusions

This case of a purely epidural thoracic WHO Grade 1 meningioma with an elevated Ki-67 index represents a novel contribution to the limited literature on extradural spinal meningiomas. It challenges conventional assumptions about the indolent nature of Grade 1 lesions and underscores the potential prognostic value of proliferation markers in guiding follow-up and management. Furthermore, the patient’s history of multiple malignancies and long-term hormone replacement therapy raises important questions about the role of hormonal and genetic factors in meningioma pathogenesis. This case highlights the need for heightened diagnostic vigilance in patients with prior cancer histories and supports further investigation into the biological behavior of extradural meningiomas and their relationship to systemic risk factors.

## References

[1] Hohenberger C, Hau P, Schebesch KM (2023). Spinal meningiomas. Neurooncol Adv.

[2] Izzuddeen Y (2021). Meningioma. Evidence Based Practice in Neuro-oncology.

[3] Wang ZL, Mou JH, Sun D, Liu P (2022). Case report: upper thoracic purely extradural spinal meningioma with nerve root attachment: a case report and literature review. Front Surg.

[4] Bettaswamy G, Ambesh P, Das KK (2016). Extradural spinal meningioma: Revisiting a rare entity. J Craniovertebr Junction Spine.

[5] Garaud S, Boto J, Egervari K, Vargas MI (2022). Extradural spinal meningioma mimicking a Schwannoma: magnetic resonance imaging findings. Can J Neurol Sci.

[6] Seo EH, Cha JG, Yoon YS, Moon AR (2022). Extradural spinal lymphoplasmacyte-rich meningioma in the thoracic spine: a case report and literature review. J Korean Soc Radiol.

[7] Gader G, Masmoudi M, Ghedira K (2023). Lumbar spine epidural meningioma: report of a rare case. Spinal Cord Ser Cases.

[8] Hsieh PC, Lu JC, Huang SC (2024). Unusual clinical presentation of cervical extradural meningioma detected with neuromuscular ultrasound: a case report. Exp Ther Med.

[9] di Bonaventura R, Caccavella VM, Latour K (2023). Spinal epidural atypical meningioma: case report and review of the literature. Acta Neurochir Suppl.

[10] Liu N, Song SY, Jiang JB (2020). The prognostic role of Ki-67/MIB-1 in meningioma: a systematic review with meta-analysis. Medicine (Baltimore).

[11] Agopiantz M, Carnot M, Denis C (2023). Hormone receptor expression in meningiomas: a systematic review. Cancers.

[12] Hage M, Plesa O, Lemaire I, Raffin Sanson ML (2022). Estrogen and progesterone therapy and meningiomas. Endocrinology.

[13] Yen YS, Sun LM, Lin CL (2014). Higher risk for meningioma in women with uterine myoma: a nationwide population-based retrospective cohort study. J Neurosurg.

[14] Bhattacharya P, Leslie SW, McHugh TW (2025). Lynch Syndrome (Hereditary Nonpolyposis Colorectal Cancer). https://www.ncbi.nlm.nih.gov/books/NBK431096/.

